# Protein biomarkers and alternatively methylated cell-free DNA detect early stage pancreatic cancer

**DOI:** 10.1136/gutjnl-2023-331074

**Published:** 2023-12-13

**Authors:** Roni Ben-Ami, Qiao-Li Wang, Jinming Zhang, Julianna G Supplee, Johannes F Fahrmann, Roni Lehmann-Werman, Lauren K Brais, Jonathan Nowak, Chen Yuan, Maureen Loftus, Ana Babic, Ehsan Irajizad, Tal Davidi, Aviad Zick, Ayala Hubert, Daniel Neiman, Sheina Piyanzin, Ofer Gal-Rosenberg, Amit Horn, Ruth Shemer, Benjamin Glaser, Natalia Boos, Kunal Jajoo, Linda Lee, Thomas E Clancy, Douglas A Rubinson, Kimmie Ng, John A Chabot, Fay Kastrinos, Michael Kluger, Andrew J Aguirre, Pasi A Jänne, Nabeel Bardeesy, Ben Stanger, Mark H O'Hara, Jacob Till, Anirban Maitra, Erica L Carpenter, Andrea J Bullock, Jeanine Genkinger, Samir M Hanash, Cloud P Paweletz, Yuval Dor, Brian M Wolpin

**Affiliations:** 1 Department of Developmental Biology and Cancer Research, IMRIC, Faculty of Medicine, The Hebrew University of Jerusalem, Jerusalem, Israel; 2 Department of Medical Oncology, Dana-Farber Cancer Institute, Harvard Medical School, Boston, Massachusetts, USA; 3 Department of Clinical Science, Intervention and Technology, Karolinska Institutet, Stockholm, Sweden; 4 Department of Clinical Cancer Prevention, The University of Texas MD Anderson Cancer Center, Houston, Texas, USA; 5 Department of Pathology, Brigham and Women's Hospital, Boston, Massachusetts, USA; 6 Sharett Institute of Oncology, Hebrew University-Hadassah Medical Center, Jerusalem, Israel; 7 Department of Endocrinology and Metabolism, Hadassah Medical Center, Jerusalem, Israel; 8 Department of Medicine, Brigham and Women's Hospital, Harvard Medical School, Boston, Massachusetts, USA; 9 Department of Surgery, Brigham and Women's Hospital, Harvard Medical School, Boston, Massachusetts, USA; 10 Department of Surgery, Herbert Irving Comprehensive Cancer Center, Columbia University Medical Center, New York, New York, USA; 11 Division of Digestive and Liver Diseases, Columbia University Irving Medical Cancer and the Vagelos College of Physicians and Surgeons, New York, New York, USA; 12 Massachusetts General Hospital Cancer Center, Center for Cancer Research, Massachusetts General Hospital, Boston, Massachusetts, USA; 13 Department of Medicine, Division of Gastroenterology, Abramson Family Cancer Research Institute, University of Pennsylvania Perelman School of Medicine, Philadelphia, Pennsylvania, USA; 14 Department of Medicine, Division of Hematology-Oncology, Perelman School of Medicine, University of Pennsylvania, Philadelphia, Pennsylvania, USA; 15 Department of Pathology, The University of Texas M. D. Anderson Cancer Center, Houston, Texas, USA; 16 Division of Hematology and Oncology, Beth-Israel Deaconess Medical Center and Harvard Medical School, Boston, Massachusetts, USA; 17 Department of epidemiology, Mailman school of public health, Columbia university, New York, New York, USA; 18 Herbert Irving Comprehensive Cancer Center, Columbia university Irving Medical Center, New York, New York, USA

**Keywords:** PANCREATIC CANCER, METHYLATION, TUMOUR MARKERS

## Abstract

**Objective:**

Pancreatic ductal adenocarcinoma (PDAC) is commonly diagnosed at an advanced stage. Liquid biopsy approaches may facilitate detection of early stage PDAC when curative treatments can be employed.

**Design:**

To assess circulating marker discrimination in training, testing and validation patient cohorts (total n=426 patients), plasma markers were measured among PDAC cases and patients with chronic pancreatitis, colorectal cancer (CRC), and healthy controls. Using CA19-9 as an anchor marker, measurements were made of two protein markers (TIMP1, LRG1) and cell-free DNA (cfDNA) pancreas-specific methylation at 9 loci encompassing 61 CpG sites.

**Results:**

Comparative methylome analysis identified nine loci that were differentially methylated in exocrine pancreas DNA. In the training set (n=124 patients), cfDNA methylation markers distinguished PDAC from healthy and CRC controls. In the testing set of 86 early stage PDAC and 86 matched healthy controls, CA19-9 had an area under the receiver operating characteristic curve (AUC) of 0.88 (95% CI 0.83 to 0.94), which was increased by adding TIMP1 (AUC 0.92; 95% CI 0.88 to 0.96; p=0.06), LRG1 (AUC 0.92; 95% CI 0.88 to 0.96; p=0.02) or exocrine pancreas-specific cfDNA methylation markers at nine loci (AUC 0.92; 95% CI 0.88 to 0.96; p=0.02). In the validation set of 40 early stage PDAC and 40 matched healthy controls, a combined panel including CA19-9, TIMP1 and a 9-loci cfDNA methylation panel had greater discrimination (AUC 0.86, 95% CI 0.77 to 0.95) than CA19-9 alone (AUC 0.82; 95% CI 0.72 to 0.92).

**Conclusion:**

A combined panel of circulating markers including proteins and methylated cfDNA increased discrimination compared with CA19-9 alone for early stage PDAC.

**WHAT IS ALREADY KNOWN ON THIS SUBJECT**

- There is an unmet need for biomarkers that allow non-invasive detection of early-stage pancreatic cancer. Circulating proteins, oncogenic mutations in cfDNA and altered methylation in cfDNA are important candidate markers for PDAC early detection.

**WHAT THIS STUDY ADDS**

- A combination of plasma proteins and pancreas-specific methylation markers in cfDNA improved detection of pancreatic cancer compared to CA-19-9 and to each marker alone.

**HOW THIS STUDY MIGHT AFFECT RESEARCH, PRACTICE OR POLICY**

- A combination of protein and tissue-specific methylation cfDNA markers may allow for detection of pancreatic cancer at an earlier and curable stage.

## Introduction

Pancreatic cancer is a leading cause of cancer death worldwide.^1^ This high mortality results in large part from >80% of patients presenting with locally advanced or metastatic disease that is incurable. In contrast, patients who present with earlier stage disease can be treated with multimodality therapy and achieve long-term survival.^2 3^

Early detection of pancreatic ductal adenocarcinoma (PDAC) remains difficult. The disease causes few early warning symptoms and has few risk factors with high penetrance.^4^ Thus far, patients with a strong family history or genetic predisposition and those with pancreatic cystic lesions have been the primary focus of early detection programmes.^5 6^ These programmes predominantly use abdominal MRI and endoscopic ultrasound to serially evaluate the pancreas for the development of cancer. Blood-based early detection approaches may allow for identification of those patients who would most benefit from imaging or endoscopic procedures.^7^ Circulating carbohydrate antigen 19–9 (CA19-9) is commonly used as a marker of treatment response in PDAC, but it may also have utility as an ‘anchor’ marker on which to add further blood-based technologies for early cancer detection.^8^ Thus, we sought to develop a panel of circulating markers that included CA19-9 and could be used in a screening setting to assess asymptomatic individuals for pancreatic cancer.

Recent studies have evaluated mutations in cell-free DNA (cfDNA) in plasma as a biomarker for the presence of early cancer.^9^ This approach has appeal for detection of pancreatic cancer, as ~90% of patients with this malignancy have point mutations in the oncogene *KRAS*.^10 11^ Nevertheless, detection of driver gene mutations in cfDNA has thus far had modest sensitivity for early stage malignancies. Furthermore, identification of a mutation in a gene such as *KRAS* does not identify the tissue origin of malignancy, as mutated genes are shared across tumour types. Oncogenic mutations in cfDNA may also reflect non-malignant conditions, such as clonal haematopoiesis.^12^ A potential approach to increase sensitivity for detection of early cancers and assist in determining the malignant site of origin leverages tissue-specific DNA methylation patterns.^13–15^ The methylation of cytosines adjacent to guanines (CpG sites) is an essential determinant of gene expression and can serve as a definitive marker of cell identity.^16^ Therefore, cfDNA molecules derived from genomic loci with tissue-specific methylation patterns can be used to identify the relative contribution of specific cell types to cfDNA and estimate the rate of cell death in specific tissues.^13^ Since tissue-specific methylation markers are largely maintained on oncogenic transformation, evaluation of cfDNA methylation can provide a powerful tool to detect and determine the tissue of origin for a growing cancer.^17^ Recently, several studies have examined altered methylation of circulating cfDNA for detection of single cancer types or for multicancer detection.^17–19^

Given the potential utility of adding cfDNA assays to multimarker panels for asymptomatic PDAC detection, we evaluated three protein markers (CA19-9, TIMP1, LRG1),^20^ *KRAS* mutation from cfDNA, and exocrine pancreas-specific methylation markers from cfDNA in PDAC cases and controls. In over 400 patients, we demonstrate the utility of combining protein markers with measures of cfDNA tissue-specific methylation in detecting early stage PDAC.

## Methods

### Study populations

**Figure 1:**
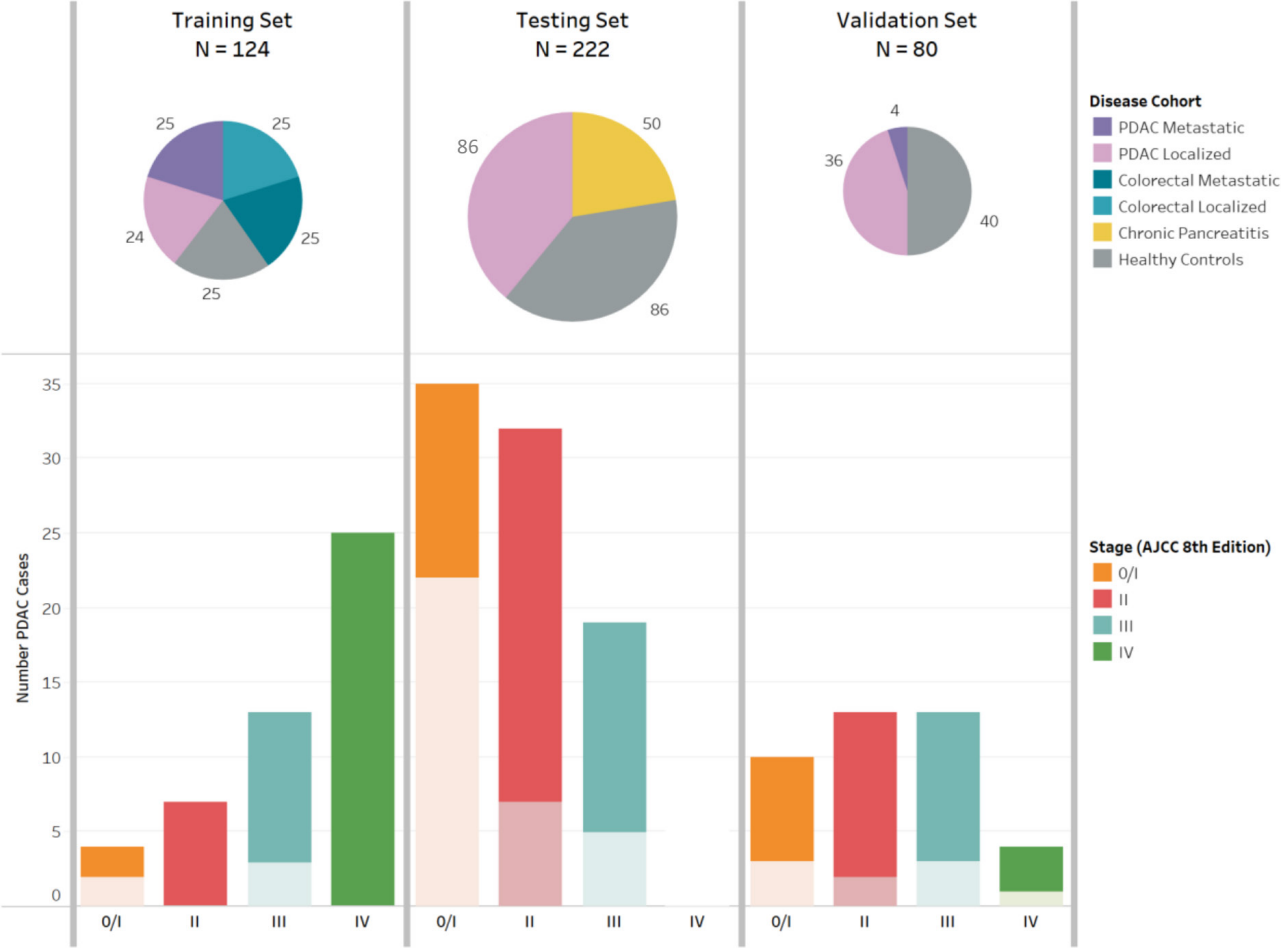
Training, testing and validation sets for characterisation of protein and cell-free DNA markers to detect early stage pancreatic cancer. AJCC, American Joint Committee on Cancer; cfDNA, cell-free DNA; CRC, colorectal cancer; PDAC, pancreatic ductal adenocarcinoma In the bar plot, lighter color shade indicates receipt of neoadjuvant therapy prior to pathologic staging among patients who went to the operating room for surgical resection. All blood samples collected at the time of cancer diagnosis prior to any treatment or surgery. Circulating markers measured: (a) Training set: cfDNA mutation and methylation; (b) Testing set: cfDNA mutation and methylation, CA19-9, TIMP1, and LRG1; (c) Validation set: cfDNA methylation, CA19-9, TIMP1, and LRG1.

Training, testing and validation case-control sets were identified or enrolled with collection of clinical data and biospecimens (figure 1). For the *training set*, we identified 125 patients with PDAC and colorectal cancer (CRC), and healthy controls treated at Dana-Farber/Brigham and Women’s Cancer Center (DF/BWCC) between 2010 and 2017. Patients with cancer had no prior cancer-directed treatment, except one patient with localised PDAC who was excluded due to blood collection after surgical resection. The final cohort included 24 with localised PDAC, 25 with metastatic PDAC, 25 with localised CRC, 25 with metastatic CRC and 25 healthy controls. Institutional, Clinical Laboratory Improvement Amendments (CLIA)-certified DNA sequencing of matched tumour DNA was available for all patients, except one with metastatic CRC whose sequencing could not be completed due to low tumour cellularity.

For the *testing set*, we prospectively accrued 86 patients with previously untreated localised PDAC who underwent subsequent surgical resection, 86 healthy controls matched by age and sex, and 50 patients with chronic pancreatitis, enrolled between 2015 and 2019 at three institutions (DF/BWCC, Beth Israel Deaconess Medical Centre and Columbia University-Irving Cancer Centre). Healthy controls had no history of cancer 5 years prior to sample collection and were recruited at the time of a screening colonoscopy or when accompanying a non-blood-related relative to the GI cancer clinic. Patients with chronic pancreatitis were identified in specialty gastroenterology clinics and aetiology was identified by medical record review. For the *validation set*, we enrolled another prospectively accrued 40 patients with previously untreated, localised PDAC and 40 age-matched and sex-matched healthy controls from the University of Pennsylvania between 2016 and 2021. Healthy controls were recruited at the time of screening colonoscopy. All patients provided informed consent for access to medical records and blood samples. Medical record review identified clinical data and tumour characteristics. Blood samples collected prior to therapy were processed to EDTA plasma within 2–3 hours and aliquots were stored at −80°C. A portion of samples from the validation set was processed in Streck tubes rather than EDTA plasma. Participant identity was blinded to laboratory personnel.

### Circulating cfDNA mutation and methylation assays

Droplet digital PCR (ddPCR) and next generation sequencing (NGS) for *KRAS* and other genes were performed as previously described (online supplemental methods, table 1).^21^ To identify exocrine pancreas-specific cfDNA markers (online supplemental table 2), we performed comparative analysis of human tissue and cell type methylomes from public sources and methylomes generated from freshly isolated, sorted cells from surgical material, using whole genome bisulfite sequencing.^22^ CpG sites found to be uniquely methylated or unmethylated were selected as potential markers distinguishing cfDNA from the exocrine pancreas. For each candidate CpG, we verified that it retained its methylation pattern in the TCGA collection of methylomes from PDAC and other tumours. To maximise tissue specificity of methylation patterns we leveraged the regional nature of DNA methylation and defined a marker as a genomic locus of <150 bp (typical nucleasome size of cfDNA fragments) that contains at least four CpGs in addition to the identified anchor site. A molecule was assigned pancreas origin when all CpGs within it had a homogenous methylation pattern consistent with that seen in exocrine pancreas. cfDNA was treated with bisulfite, PCR-amplified in multiplex and sequenced as described (online supplemental methods).^23^ To correct for the presence of cfDNA derived from other tissues, we multiplied the fraction of pancreas-specific molecules by the total concentration of cfDNA in each sample to provide concentration of exocrine pancreas-specific cfDNA in a sample, expressed as pancreas genome equivalents per mL plasma. Pancreas cfDNA signal was calculated as the average signal obtained from the multiple markers.

10.1136/gutjnl-2023-331074.supp1 Supplementary data

10.1136/gutjnl-2023-331074.supp2 Supplementary data

### Protein biomarkers

Plasma protein concentrations for CA19-9, LRG1 and TIMP1 were measured by bead-based ELISA using Luminex multiplexed assay technology, as previously described.^20^ This approach was used to minimise required sample volume for research purposes and was not for diagnostic application.

### Statistical analysis

Clinical characteristics were described using mean (SD) for continuous variables and number (percent) for categorical variables. Receiver operating characteristic (ROC) curves were generated using logistic regression with calculation of area under the ROC curve (AUC) to provide a measure of model discrimination.^24^ Confidence limits for AUC were calculated by the Wald method. Comparisons of differences between AUCs were tested using Delong’s non-parametrical approach.^25^ Sensitivity was reported at ≥90%, ≥95% and≥98% specificity, given the desire to limit false-positive results when detecting a malignancy of low incidence. Assay cut points for positivity were determined from ≥98% specificity in the testing set.

### Patient and public involvement

Patients were not directly involved in conduct of the study; however, the public has strongly advocated for advancements in PDAC early detection.

## Results

### Methylation markers of the exocrine pancreas

**Figure 2:**
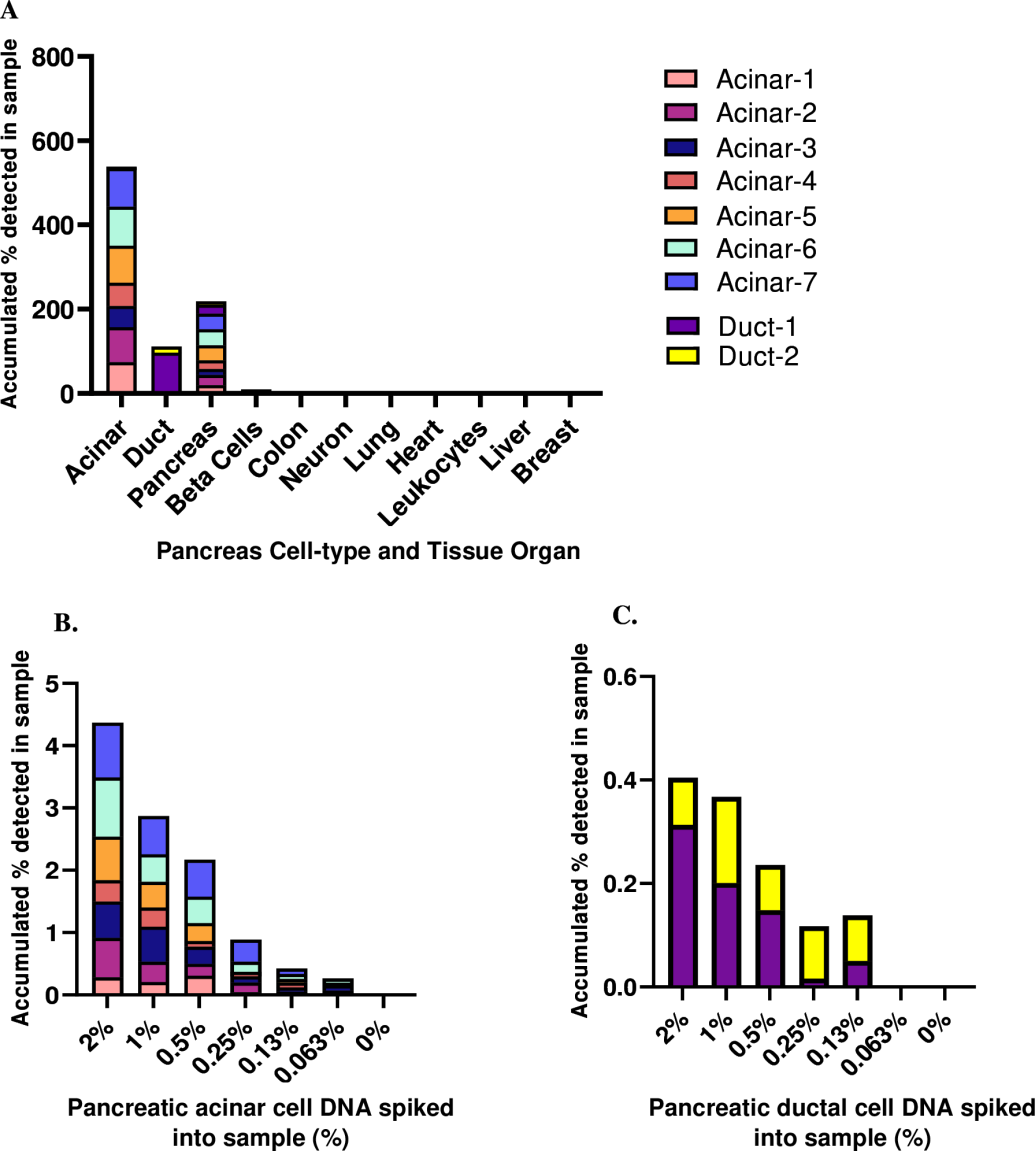
Tissue specificity and spike-in sensitivity of exocrine pancreas methylation markers for pancreas acinar and ductal cells. Methylation status of acinar and duct-derived markers in genomic DNA from multiple human tissues (A). Each color represents a locus that is differentially methylated or unmethylated in a specific cell type. Shown is the methylation score of multiple CpG sites in each block (i.e. the fraction of molecules that are fully methylated or unmethylated in a given sample). Sensitivity of acinar-derived (B) or duct-derived (C) methylation markers. Pancreas-specific DNA was spiked into leukocyte DNA as indicated and the fraction of pancreas DNA was assessed using bisulfite conversion, multiplex PCR amplification of acinar markers and sequencing.

Comparative methylome analysis resulted in the identification of two loci that were specifically methylated in pancreatic acinar cells (termed acinar-1 and acinar-2). To validate specificity and examine sensitivity, we amplified these loci from bisulfite-treated genomic DNA derived from a panel of human tissues and sequenced the products. Each marker was fully methylated in 74%–83% of DNA from acinar cells, whereas no fully methylated molecules were identified in leucocytes or other tissues (figure 2A). To assess marker sensitivity, we spiked acinar genomic DNA into leucocyte DNA in known proportions and measured the signal obtained after PCR and sequencing. We detected the presence of as little as 0.1% pancreas DNA in the mixture (figure 2B). Moreover, we verified that these markers retained their altered methylation patterns in PDAC (online supplemental figure 1). Due to modest sensitivity for localised PDAC with two markers in the training set (see below), we expanded our marker set to include seven additional loci differentially methylated in acinar cells (acinar-3 to acinar-7) or ductal epithelial cells (duct-1, duct-2) for the testing and validation sets. All seven new markers showed high organ specificity, spike-in experiments demonstrated identification of exocrine pancreas DNA when comprising as little as 0.1% of DNA in a mixture (figure 2), and these markers retained their methylation patterns in PDAC (online supplemental figure 1). We further verified specificity compared with other cancer types (online supplemental figure 2).

### Training set

We investigated cfDNA mutation and methylation in 124 patients with PDAC and CRC, and healthy controls (online supplemental tables 3,4). The cfDNA sequencing for *KRAS* mutations identified 21% of localised PDAC, 72% of metastatic PDAC, 12% of localised CRC, 36% of metastatic CRC and 4% of healthy controls (online supplemental figure 3A). In an exploratory analysis of amplicon-based NGS, including for *KRAS, TP53, GNAS, SMAD4, RNF43, CDKN2A* and *BRAF,* sensitivity was not improved, and more false-positive results were identified (online supplemental table 4). Therefore, this approach was not pursued in the testing set.

We next examined exocrine pancreas-specific cfDNA by bisulfite sequencing of the acinar-1 and acinar-2 loci in the training set (online supplemental figure 3B). Comparing PDAC cases (n=49) to CRC cases and healthy controls (n=75), the AUC by ROC curve analysis was 0.77, with sensitivity for PDAC detection of 57% at 95% specificity, and 55% at 98% specificity. When considered by stage at diagnosis with 98% specificity, sensitivity was 29% for localised PDAC and 80% for metastatic PDAC.

### Testing set

Given the ability to detect PDAC with circulating cfDNA approaches in the training set, we next examined the testing set that included 86 patients with localised PDAC, 86 healthy controls and 50 patients with chronic pancreatitis (online supplemental tables 5,6). Although chronic pancreatitis is rare in the general population, this patient group was included to assess the specificity of markers in the context of an inflammatory condition of the pancreas. Given the potential role of CA19-9 as an anchor marker for PDAC detection,^8^ we first measured CA19-9 in the testing set cases and controls (table 1). CA19-9 had an AUC of 0.89 (95% CI 0.84 to 0.94) comparing patients with localised PDAC to healthy controls and AUC of 0.85 (95% CI 0.79 to 0.91) comparing patients with localised PDAC to those with chronic pancreatitis.

**Table 1:** Discrimination of patients with early stage pancreatic cancer, healthy controls and patients with chronic pancreatitis by protein and cell-free DNA markers in the testing set

*Biomarker*	No. cases	No. controls	AUC (95% CI)	Sensitivity at designated specificities		
				≥90%	≥95%	≥98%
Local PDAC versus healthy controls						
CA19-9	86	86	0.89 (0.84 to 0.94)	67%	67%	64%
TIMP1	86	86	0.83 (0.77 to 0.89)	59%	38%	19%
LRG1	86	86	0.79 (0.73 to 0.86)	50%	36%	31%
cfDNA *KRAS* mutation	86	86	0.54 (0.50 to 0.57)	9%	9%	9%
2-loci cfDNA methylation panel*	84	82	0.55 (0.49 to 0.61)	18%	13%	11%
9-loci cfDNA methylation panel*†*	84	82	0.69 (0.61 to 0.77)	40%	33%	21%
Local PDAC versus chronic pancreatitis						
CA19-9	86	50	0.85 (0.79 to 0.91)	67%	64%	52%
TIMP1	86	50	0.68 (0.59 to 0.78)	38%	17%	13%
LRG1	86	50	0.68 (0.59 to 0.78)	27%	12%	12%
cfDNA *KRAS* mutation	86	50	0.53 (0.48 to 0.57)	9%	9%	2%
2-loci cfDNA methylation panel*	84	48	0.58 (0.53 to 0.63)	21%	19%	17%
9-loci cfDNA methylation panel†	84	48	0.69 (0.60 to 0.78)	40%	24%	7%

*2-loci cfDNA methylation panel that includes two exocrine pancreas loci encompassing 17 CpG sites.

†9-loci cfDNA methylation panel that includes nine exocrine pancreas loci encompassing 61 CpG sites.

AUC, area under the receiver operating characteristic curve; cfDNA, cell-free DNA; PDAC, pancreatic ductal adenocarcinoma.

Given the potential for protein markers to increase sensitivity when combined with cfDNA detection,^26 27^ we measured plasma TIMP1 and LRG1, which we previously identified as protein biomarkers for early stage PDAC (table 1). TIMP1 had an AUC of 0.83 (95% CI 0.77 to 0.89) comparing patients with early stage PDAC to healthy controls and AUC of 0.68 (95% CI 0.59 to 0.78) when compared with patients with chronic pancreatitis. Plasma LRG1 had an AUC of 0.79 (95% CI 0.73 to 0.86) when patients with early stage PDAC were compared with healthy controls and AUC of 0.68 (95% CI 0.59 to 0.78) when compared with patients with chronic pancreatitis.

We next evaluated cfDNA mutation and methylation detection in the testing set (table 1). Among cfDNA detection approaches, the highest AUC for the comparisons of localised PDAC to both healthy controls and chronic pancreatitis was identified for the cfDNA methylation approach using nine methylation haplotype blocks, with AUC of 0.69 in comparison to both control groups. Since greater sensitivity may be achieved with the combination of several markers, we next examined whether the protein and cfDNA markers provided additional discrimination beyond CA19-9 alone for cases and controls. The AUC for discrimination of early stage PDAC from healthy controls increased with addition of TIMP1, LRG1 or the 9-loci cfDNA methylation panel to CA19-9 (table 2), but not with the addition of cfDNA *KRAS* mutation or the 2-loci cfDNA methylation panel. Little benefit in AUC was identified for these markers when added to CA19-9 in discriminating early stage PDAC from patients with chronic pancreatitis (table 2).

**Table 2:** Discrimination of patients with early stage pancreatic cancer, healthy controls and patients with chronic pancreatitis by protein and cfDNA markers when added to CA19-9 in the testing set

Biomarker	No. cases	No. controls	AUC (95% CI)	*P* value	Sensitivity at designated specificities		
					≥90%	≥95%	≥98%
**Local PDAC versus healthy controls**							
CA19-9	84	82	0.88 (0.83 to 0.94)	Reference	67%	67%	63%
+ TIMP1	84	82	0.92 (0.88 to 0.96)	0.06	80%	75%	63%
+ LRG1	84	82	0.92 (0.88 to 0.96)	0.02	77%	77%	68%
+ cfDNA *KRAS* mutation	84	82	0.88 (0.83 to 0.94)	0.39	67%	67%	63%
+ 2-loci cfDNA methylation panel*	84	82	0.89 (0.83 to 0.94)	0.56	67%	67%	63%
+ 9-loci cfDNA methylation panel†	84	82	0.92 (0.88 to 0.96)	0.02	76%	70%	65%
**Local PDAC versus chronic pancreatitis**							
CA19-9	84	48	0.85 (0.78 to 0.91)	Reference	67%	63%	52%
+ TIMP1	84	48	0.84 (0.78 to 0.91)	0.88	67%	55%	48%
+ LRG1	84	48	0.85 (0.79 to 0.91)	0.51	68%	60%	57%
+ cfDNA *KRAS* mutation	84	48	0.85 (0.78 to 0.91)	1.00	67%	63%	52%
+ 2-loci cfDNA methylation panel*	84	48	0.86 (0.79 to 0.92)	0.19	68%	62%	55%
+ 9-loci cfDNA methylation panel†	84	48	0.86 (0.80 to 0.92)	0.35	67%	56%	54%

*2-loci cfDNA methylation panel that includes two exocrine pancreas loci encompassing 17 CpG sites.

†9-loci cfDNA methylation panel that includes nine exocrine pancreas loci encompassing 61 CpG sites.

AUC, area under the receiver operating characteristic curve; cfDNA, cell-free DNA; PDAC, pancreatic ductal adenocarcinoma.

**Figure 3:**
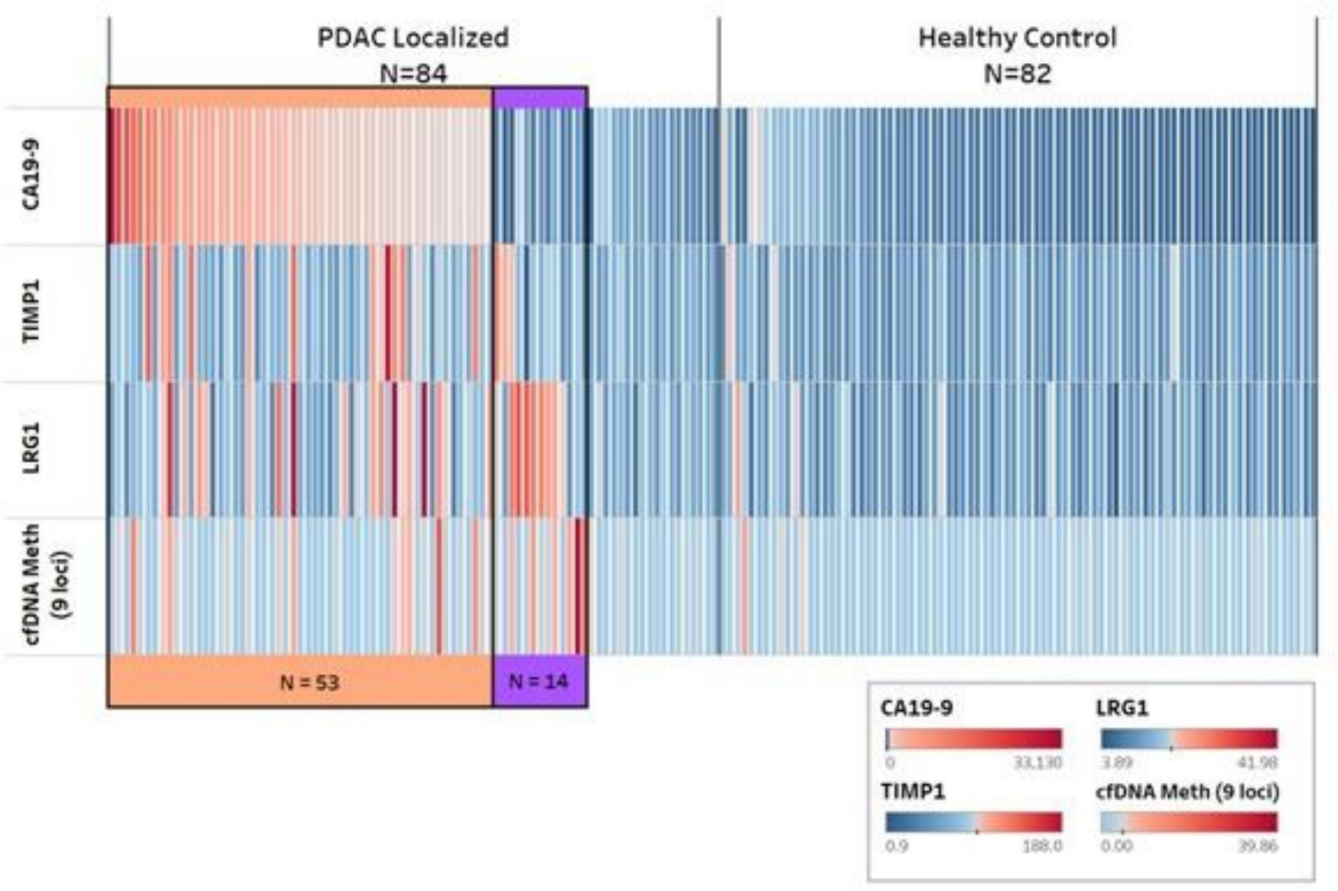
Cumulative positivity for early stage pancreatic cancer and healthy controls by protein and cell-free DNA markers in the testing set. cfDNA, cell-free DNA; PDAC, pancreatic ductal adenocarcinoma Each column represents one subject and each row represents their value for the designated biomarker, with red bar indicating positive and blue bar indicating negative value by heatmap scale. Cases and controls are each sorted from highest to lowest CA19-9 values for CA19-9 positive cases and then in order by positive values for TIMP1, LRG1, and 9-loci cfDNA methylation panel. Orange track color indicates detected cases by CA19-9. Purple track color indicates detected cases by other markers among CA19-9 negative cases.

We also considered the performance of the protein and cfDNA markers among cases identified as CA19-9-negative (figure 3). Among the 31 patients without CA19-9 elevation, TIMP1 was elevated in 3 cases, LRG1 in 8 cases and the 9-loci cfDNA methylation panel in 7 cases, when considering cut points for positivity that conveyed ≥98% specificity for the individual marker. When considering these markers together, 14 (45%) of the 31 CA19-9-negative cases were positive for one or more of these markers, indicating the presence of an early stage PDAC among cases without elevated CA19-9. We then constructed several multimarker panels with CA19-9 as the anchor marker and including combinations of the two protein markers and the 9-loci cfDNA methylation panel. All panels performed similarly in the testing set with AUCs of 0.94, which were statistically significant improvements over the AUC of 0.88 with CA19-9 alone (p<0.05 for all models; online supplemental table 7).

### Validation set

**Figure 4:**
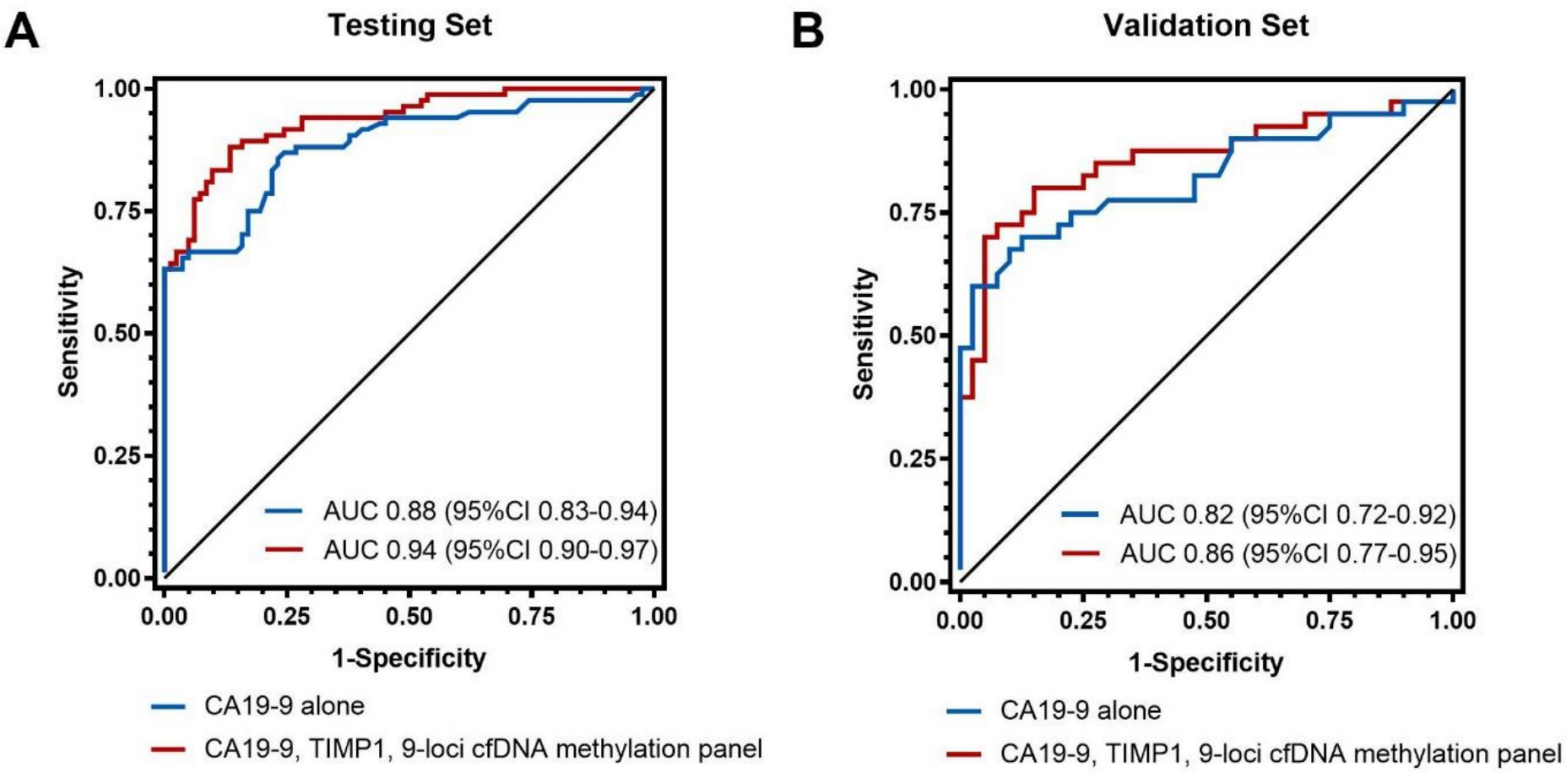
Receiver operating characteristic curves for plasma CA19-9 alone and in combination with TIMP1 and the 9-loci cfDNA methylation panel for distinguishing early stage pancreatic cancer from healthy controls in the testing (A) and validation (B) sets. AUC, area under the receiver-operator characteristic (ROC) curve; cfDNA, cell-free DNA.

In an independent validation set including 40 patients with PDAC and 40 healthy controls (online supplemental tables 8,9), we looked to validate the four prediction models combining proteins and the 9-loci cfDNA methylation panel. We first evaluated the discrimination of individual biomarkers in the external validation set (online supplemental table 10). Compared with AUC values in the testing set, LRG1 performed substantially less well in the validation set, while the other three markers had similar AUC, including CA19-9 (AUC, 0.82; 95% CI 0.72 to 0.92), TIMP1 (AUC, 0.76; 95% CI 0.65 to 0.87) and the 9-loci cfDNA methylation panel (AUC, 0.69; 95% CI 0.58 to 0.81). We subsequently examined discrimination of the multimarker panels with fixed model coefficients calculated from the testing set (figure 4, online supplemental table 7). The fixed model containing CA19-9, TIMP1 and the 9-loci cfDNA methylation panel (AUC, 0.86; 95% CI 0.77 to 0.95) had a higher AUC than CA19-9 alone (AUC, 0.82; 95% CI 0.72 to 0.92).

## Discussion

Diagnosis of early stage disease greatly improves the chance of long-term survival of patients with PDAC. Liquid biopsies for molecular characterisation and disease follow-up during treatment have entered clinical care,^28^ and large studies are now being pursued to apply liquid biopsies to early cancer detection.^15 29^ However, complementary technologies will likely be needed to identify early cancers with high sensitivity, and optimal marker combinations may differ by cancer type. Here, we demonstrate improved sensitivity when protein and cfDNA markers are added to CA19-9, a potential anchor marker on which to build multimarker detection approaches for PDAC.^8^

We evaluated the performance of circulating markers in the training and testing cohorts and then assessed additive discriminatory capacity for early stage disease in combination with plasma CA19-9 in an independent validation cohort. Although accepted as a prognostic marker for pancreatic cancer, CA19-9 is not routinely used in the screening setting. However, among patients in the testing and validation sets of the current study, the AUC was 0.82–0.89 comparing PDAC cases to healthy controls, and sensitivity was ≥60% at a specificity of ≥98%. These discriminatory statistics suggest that CA19-9 may function satisfactorily as an anchor marker on which to add further early detection technologies. Additionally, genotyping inherited genetic variants may further increase CA19-9 performance, given the ~10% of individuals who do not synthesise CA19-9 due to biallelic inactivating polymorphisms in *FUT3*.^30^ Nevertheless, it is important to note that differences between CA19-9 assays can complicate threshold selection and cross-study comparisons,^31^ and studies of prediagnosis plasma suggest that elevations are likely to occur predominantly in the 6–12 months prior to cancer diagnosis,^8 32^ necessitating relatively frequent measurements to capture patients early in the disease process. In addition, the performance of CA19-9 as a potential anchor marker may vary among screening populations, including those at high risk and in the general population.

Although CA19-9 serves as a candidate marker for PDAC early detection, a sizeable number of patients will not be identified by using CA19-9 alone. In the current study, patients with CA19-9 below the positive threshold were identified by assessment of additional protein markers (TIMP1 and LRG1) or exocrine pancreas-specific methylated cfDNA, suggesting the complementarity of different markers for PDAC detection. More than 90% of PDACs have a point mutation in the *KRAS* oncogene,^10 11^ suggesting this mutation as a cfDNA marker for early detection. Nevertheless, studies thus far have indicated modest sensitivity for *KRAS* mutation detection in early stage localised PDAC,^26 27 33 34^ and need for large plasma volumes to detect rare tumour DNA fragments. In the current study, multiplexed ddPCR for *KRAS* codons 12 and 61 identified only 10% of patients with early stage PDAC at 98% specificity in the testing cohort. In contrast, the large majority of patients with metastatic disease were identified in the training cohort. Alternative high-sensitivity detection approaches or the use of larger amounts of plasma may improve on these results, but cfDNA *KRAS* mutation detection did not provide additive information with plasma CA19-9 in the current study.

Recurrent mutations in PDAC are identified primarily in *KRAS* and *TP53*, limiting the areas of the cfDNA genome that are informative for early disease detection. In contrast, many pancreas-specific methylation markers are conserved in the genome, potentially enhancing the detection of rare DNA fragments originating from cancer. Furthermore, given the organ specificity of methylation markers, the tissue of origin may be inferred within the same assay platform, potentially helping to guide clinical evaluation.^14 15 35^ Notably, false-positive test results due to clonal haematopoiesis are also reduced with methylation-based approaches that measure organ-specific cfDNA fragments compared with detection of mutations.^36 37^ In the current study, pancreas-specific methylation markers added discriminatory capacity beyond CA19-9 for early stage disease, but only with our larger panel of methylation blocks. Further increases in the number of pancreas-specific markers may facilitate even greater assay sensitivity.^38 39^ However, the use of a relatively small number of methylation markers harbouring very high pancreas specificity allows for measurement of essentially all DNA molecules containing each marker sequence in a sample (ie, coverage of >1000 ×), potentially providing higher sensitivity for detection of pancreas cfDNA at a lower cost.

Previous studies have identified methylation or hydroxymethylation changes in PDAC and then evaluated their occurrence in cfDNA,^40–42^ but these methylation changes were not exclusive to pancreatic cancer compared with other tumour types or well conserved across pancreatic cancers. In the current study, we identified methylation signatures of the normal exocrine pancreas that were preserved in a large cohort of pancreatic cancers. This approach was designed to facilitate high specificity, while also enhancing sensitivity due to evaluation of methylation signatures common across pancreatic tumours and measurement of the joint effect of cell death of tumour cells and adjacent normal cells. Although this approach could theoretically detect non-malignant pancreatic pathologies, we did not identify higher levels of pancreas-derived cfDNA in patients with chronic pancreatitis, possibly due to the natural slow-progressive course of the disease. This finding is seemingly in contrast to our previously published work,^43^ in which patients with chronic pancreatitis had elevated levels of pancreas-derived cfDNA. In the previous work, plasma samples were obtained from patients with severe chronic pancreatitis who were hospitalised and required surgical intervention. In our current study, samples were obtained in the outpatient clinic in the absence of an acute flare. We believe this is the source of the conflicting findings. Additional studies in patients with acute pancreatitis or benign biliary disease would be informative, although these conditions are readily diagnosed by clinical and laboratory evaluation, so unlikely to reduce test specificity in a screening population.

The addition of TIMP1 and circulating methylated cfDNA to CA19-9 increased PDAC discrimination; however, a combined marker approach can increase the number of false-positive results. Thus, the clinical utility of combining additional markers with CA19-9 will be dependent on the population for evaluation and the false-positive rate deemed tolerable. Notably, multianalyte assays may need to be tuned differently to interrogate the general population versus the high-risk groups, such as those with family history of PDAC, pancreatic cystic lesions or recent-onset diabetes,^44 45^ and future decisions regarding threshold values for a positive test will need to be made with the intended use population in mind.

The current study has a number of important strengths. The subject populations were drawn from multiple institutions using unified sample collection and processing protocols. Multiple patient groups were evaluated in training, testing and validation sets, including subjects with PDAC, CRC, chronic pancreatitis and healthy controls. The testing and validation sets were prospectively collected and included only patients with an initial diagnosis of early stage disease, constituting an important target population for early disease identification. Plasma CA19-9 was used as an anchor marker in the testing and validation sets, with the utility of further markers assessed with respect to their additive discriminatory capacity. Laboratory personnel were blinded to the case-control status of study participants, and analyses were conducted centrally using a prespecified analysis plan.

The study also has limitations that deserve consideration. Sensitivities for our cfDNA approaches in the testing and validation sets were somewhat lower than anticipated.^15 26 27^ Since circulating tumour DNA is thought to be proportional to tumour burden,^33 46^ the fact that our population was heavily skewed towards patients with early stage disease likely resulted in fewer tumour DNA fragments in the blood of these patients. Our ability to detect tumour cfDNA fragments may also have been affected by the 2 mL volume of plasma used for our cfDNA studies. Larger volumes of plasma may be helpful to identify very early stage tumours. The increased sensitivity with our greater multiplexing of methylation sites also suggests that evaluating larger areas of the genome may assist in detecting these rare tumour-derived cfDNA fragments. We evaluated our cfDNA methylation panels in patients with PDAC, CRC, chronic pancreatitis and healthy controls. Additional studies to evaluate the specificity of our protein and cfDNA methylation markers in blood for PDAC compared with other cancer types will be necessary. All blood samples were collected at the time of cancer diagnosis. As we described recently with protein markers,^8^ it is critical to define the timeframe during which circulating markers are detectable before a cancer would be diagnosed clinically, such that screening intervals can be rationally designed. The current study did not evaluate all possible early detection technologies and other promising approaches, such as genome-wide cfDNA fragmentation and circulating exosomes that are currently under evaluation.^33 47–51^

In summary, the combined detection of protein markers and pancreas-specific methylation in circulating cfDNA may improve discrimination for detection of early stage, localised PDAC compared with plasma CA19-9 alone. Additional studies are needed to determine whether this and other abovementioned approaches can lead to diagnosis of asymptomatic early stage PDAC in the general population or high-risk individuals and reduce mortality from this highly lethal malignancy.

## Data Availability

Data are available upon reasonable request. All data relevant to the study are included in the article or uploaded as supplementary information.
