# Supplementary material for: Protein biomarkers and alternatively methylated cell-free DNA detect early stage pancreatic cancer
Source: Gut. 2023 Dec 13;73(4):639–48. doi: 10.1136/gutjnl-2023-331074 (PMC10958271; doi:10.1136/gutjnl-2023-331074)
Supplement: Supplementary data [file gutjnl-2023-331074supp001.pdf]

SUPPLEMENTAL MATERIALS

<u>Supplemental Methods</u>

Circulating Cell-free DNA Extraction

Study participants in training and testing cohorts had 4ml of plasma sent to the Translational Research Laboratory of the Belfer Center for Applied Cancer Science at DFBCC, and cfDNA was extracted from plasma aliquots using Qiagen's Circulating Nucleic Acids kit. All samples were eluted in 100ul buffer AE. Eluent was aliquoted and stored at -20°C until use for: (1) ddPCR (25ul) and NGS (25ul) in the training set and ddPCR alone (50ul) in the testing set, and (2) bisulfite sequencing for methylation analysis (50ul) in all sets. For the validation set, plasma cfDNA was extracted at University of Pennsylvania by Qiagen MinElute and was eluted in 60 ul water for bisulfite sequencing for methylation analysis only (50ul).

Droplet Digital PCR for Circulating cfDNA

ddPCR for *KRAS* was performed as described.[1] Briefly, custom Taqman probes were either conjugated with VIC (wild type) or FAM (mutant) fluorophores (Applied Biosystems; **Supplemental Table 1**). For the training set, *KRAS* p.G12A, p.G12C, and p.G13D and *KRAS* p.G12D, p.G12S, and p.G12V were multiplexed in 2 ddPCR reactions. For the testing set, *KRAS* p.G12D, p.G12V, and p.G12C and *KRAS* p.Q61H, p.Q61R, and p.Q61L were multiplexed in 2 ddPCR reactions, while *KRAS* p.G12R was run as a separate single-plex assay. 10ul of cfDNA eluent (equivalent to 400ul plasma) was assayed in the training set, and 15ul of cfDNA eluent (equivalent to 600ul plasma) was assayed in the testing set. Droplets were generated using the Bio-Rad Automated Droplet Generator. ddPCR cycling conditions were 10 min at 95°C, followed by 40 cycles of a two-step thermal profile of 15s at 94°C denaturation and 60s at 60°C annealing, followed by 10°C hold. Droplets were then read on a Bio-Rad QX100 or QX200 droplet reader, and the results were analyzed using QuantaSoft.

Next-Generation Sequencing for cfDNA mutations

In the training set, target specific primers for a QIAseq Targeted DNA panel covering all exons for *KRAS, TP53, GNAS, SMAD4, RNF43, CDKN2A*, and *BRAF* were custom designed. 16.75uL cfDNA was used to generate libraries except for highly concentrated samples, in which case 80ng cfDNA was used. Samples were batched and barcoded in groups of 16 and sequenced using Illumina's NextSeq 500 and QIAseq sequencing primers. FASTQ files were aligned using Qiagen's online Data Analysis Center (https://www.qiagen.com/us/shop/genes-and-pathways/data-analysis-center-overview-page/), and BAM files were examined using IGV_2.4.10. Variants were considered true if at least three full, high-quality (>=2 reads and free of obvious PCR abnormalities) molecular barcodes contained the variant.

Pancreas-specific cfDNA Methylation Markers

To identify exocrine pancreas-specific cfDNA markers (**Supplemental Table 2**), we performed comparative analysis of a large atlas of human tissue and cell type methylomes, based on public sources (e.g. the TCGA, human roadmap epigenomic) and methylomes generated locally from freshly isolated, sorted cells from surgical material, using the Illumina Infinium HumanMethylation450, MethylationEPIC BeadChip arrays, or whole genome bisulfite sequencing.[2,3] CpG sites found to be uniquely methylated or unmethylated in pancreatic acinar or ductal cells were selected as potential markers distinguishing cfDNA from the exocrine pancreas. For each candidate CpG we verified that it retained its methylation pattern in the TCGA collection of methylomes from pancreatic cancer and other tumors. To maximize tissue specificity of methylation patterns we took advantage of the regional nature of DNA methylation

and defined a marker as a genomic locus of <150bp (considering the typical nucleasome size of cfDNA fragments) that contains at least 4 CpG sites in addition to the identified anchor site. A molecule was assigned pancreas origin when all CpG sites within it had a homogenous methylation pattern consistent with the pattern seen in exocrine pancreas. The use of methylation blocks in cfDNA analysis reduces background signal, i.e. assignment of pancreas origins to DNA derived from other tissue.[3,4]

cfDNA methylation analysis

Extracted cfDNA was treated with bisulfite, PCR-amplified in multiplex and sequenced as described.[5] The primary sequencing data for each marker report the fraction of molecules that carry that pancreas-specific methylation signature. To correct for the presence of cfDNA derived from other tissues, we multiplied the fraction of pancreas-specific molecules by the total concentration of cfDNA in each sample. This provided the concentration of exocrine pancreas-specific cfDNA in a sample, expressed as pancreas genome equivalents (GE) per ml plasma.

In the training set, we assessed two loci specifically methylated in pancreatic acinar cells as well as in PDAC (acinar-1 and acinar-2), each containing a block of 7-10 CpG sites. The pancreas cfDNA signal was calculated as the average of the signal obtained from the two markers. In the testing and validation set, we assessed these two loci, along with 7 additional methylation blocks marking the exocrine pancreas (5 acinar and 2 ductal), each uniquely methylated or unmethylated in exocrine pancreas, based on the hypothesis that adding additional markers would provide greater sensitivity for PDAC detection. We defined the levels of pancreas-specific cfDNA as the average of signal from the 9 markers expressed in exocrine pancreas.

Tumor DNA Sequencing

Tumor sequencing was performed by one of two types of CLIA-certified, institutional sequencing platforms at DF/BWCC. OncoPanel is a hybrid-capture and massively parallel sequencing assay for mutation, insertion/deletion, copy number and structural alteration detection.[6] OncoMap was a mass spectrometric genotyping platform for point mutation detection.[7]

Supplemental Tables

Supplemental Table 1. Taqman probes for KRAS mutation detection in cell-free DNA using droplet digital polymerase chain reaction

Supplemental Table 2. Genomic coordinates of exocrine-pancreatic cell type-specific methylation markers, and primer sequences used to amplify these loci after bisulfite conversion.

Supplemental Table 3. Clinical characteristics of pancreatic cancer cases and controls in training set

Supplemental Table 4. Patient characteristics and circulating cell-free DNA results for training set participants

Supplemental Table 5. Clinical characteristics of pancreatic cancer cases and controls in testing set

Supplemental Table 6. Patient characteristics and circulating marker results for testing set participants

Supplemental Table 7. Discrimination of early-stage pancreatic cancer patients and healthy controls by multi-marker panels in the testing and validation sets

Supplemental Table 8. Clinical characteristics of pancreatic cancer cases and matched controls in the validation set

Supplemental Table 9. Patient characteristics and circulating marker results for validation set participants

Supplemental Table 10. Discrimination of pancreatic cancer patients compared with healthy controls for protein and cell-free DNA methylation markers in the validation set

Ben-Ami R, *et al. Gut* 2024; 73:639–648. doi: 10.1136/gutjnl-2023-331074

Supplemental Table 1. Taqman probes for *KRAS* mutation detection in cell-free DNA using droplet digital polymerase chain reaction

See supplemental excel file

Ben-Ami R, *et al. Gut* 2024; 73:639–648. doi: 10.1136/gutjnl-2023-331074

Supplemental Table 2. Genomic coordinates of exocrine-pancreatic cell type-specific methylation markers, and primer sequences used to amplify these loci after bisulfite conversion.

Marker	Chromosome	Coordinates	Left primer	Right primer
Acinar-1	14	105714499-105714632	GGTTGATATTATAATTTGTGATAGG	CCAATCCTACTAACTAACCATATC
Acinar-2	13	32605843-32605953	TTTGTAAGGGTTGGTTGTTG	CCTACTTATTTAACCATTTACATTC
Acinar-3	16	68118294-68118398	TTTTATTTTAGATTTTAGGAGGAG	AAAAATAACACTACCTAAAAAACC
Acinar-4	12	117798054-117798157	TTATAGTGTTTTGGGGGTGG	CAAAACCACTCAAAAACCTTAC
Acinar-5	16	75263457-75263586	GTGGTTTAGTTTTTTGATTTTTTT	AACCCACTACAACAACCTACTATAC
Acinar-6	10	133806782-133806896	TTGGGATGTTTTTAGTTTTTGT	TCCATAACATTTACCTACAAAAAA
Acinar-7	16	25228628-25228773	AATTGTTGGGTTTTGTTTTTT	ATCTCACCTAATATTCCCCAAC
Duct-1	15	102157393-102157540	GAGAAAATGGTTTTAGATTATTGTA	TTAATAAATTAAAATAATATTCACCTC
Duct-2	7	156810775-156810850	AGGGGTTTTTTAGGGATA	TCCCTACTTAAACCTCAACC

Supplemental Table 3. Clinical characteristics of pancreatic cancer cases and controls in training set

Baseline Characteristics*	Pancreatic Cancer		Colorectal Cancer		Healthy Controls (N=25)
	Localized (N=24)	Metastatic (N=25)	Localized (N=25)	Metastatic (N=25)	
**Age, years**	66 (7)	63 (12)	64 (14)	55 (11)	47 (12)
**Female sex**	12 (50)	10 (40)	12 (48)	12 (48)	18 (72)
**Race/Ethnicity**					
White	24 (100)	24 (96)	24 (96)	22 (88)	21 (84)
Black	0	0	1 (4)	2 (8)	2 (8)
Other/Unknown	0	1 (4)	0	1 (4)	2 (8)
**Tobacco use**					
Current	1 (4)	1 (4)	2 (8)	0	0
Past	12 (50)	9 (36)	9 (36)	10 (40)	9 (36)
Never	11 (46)	15 (60)	13 (52)	12 (48)	11 (44)
Unknown	0	0	1 (4)	3 (12)	5 (20)
**Body-mass index, kg/m^2**	28.9 (5.0)	27.2 (5.0)	29.7 (4.8)	26.8 (5.3)	26.6 (5.6)
**Diabetes mellitus**	10 (42)	7 (29)	5 (20)	3 (12)	0
**AJCC 8th ed pTNM staging**					
Up-front surgical resection					
T0-2N0M0	2 (8)	-	5 (20)	-	-
T3-4N0M0	1 (4)	-	7 (28)	-	-
T1-4N1M0	6 (25)	-	8 (32)	-	-
T1-4N2M0	6 (25)	-	5 (20)	-	-
Neoadjuvant treatment					
T0-2N0M0	2 (8)	-	-	-	-
T3-4N0M0	0	-	-	-	-
T1-4N1M0	0	-	-	-	-
T1-4N2M0	3 (13)	-	-	-	-
Unresectable	4 (17)	-			
TxNxM1	-	25 (100)	-	25 (100)	-
**Metastatic sites**					
Liver	-	14 (54)	-	21 (84)	-
Lung	-	4 (15)	-	2 (8)	-
Peritoneum	-	8 (32)	-	3 (11)	-
Other	-	0	-	1 (4)	-
**Tumor *KRAS* mutation**					
Codon 12	18 (75)	17 (68)	5 (20)	7 (29)	-
Codon 13	0	0	3 (12)	0	-
Codon 61	4 (16)	2 (8)	1 (4)	1 (4)	-
Wild-type	2 (8)	5 (20)	15 (60)	17 (67)	-
Other†	0	1 (4)	1 (4)	0	-

Abbreviations: AJCC 8th ed pTNM staging, American Joint Committee on Cancer eighth edition tumor-node-metastasis staging

* Continuous variables reported as mean (standard deviation). Categorical variables reported as number (percent).

† One patient with metastatic pancreatic cancer with KRAS p.A66S and one patient with localized colon cancer with KRAS p.A146T.

Ben-Ami R, *et al. Gut* 2024; 73:639–648. doi: 10.1136/gutjnl-2023-331074

Supplemental Table 4. Patient characteristics and circulating cell-free DNA results for training set participants

See supplemental excel file

Ben-Ami R, *et al. Gut* 2024; 73:639–648. doi: 10.1136/gutjnl-2023-331074

Supplemental Table 5. Clinical characteristics of pancreatic cancer cases and controls in testing set

Characteristic	PDAC Cases (N=86)		Healthy Controls (N=86)		Chronic Pancreatitis (N=50)	
	No.	%	No.	%	No.	%
**Institution**						
Dana-Farber/Brigham Cancer Center	57	66%	81	94%	30	60%
Beth Israel-Deaconess Medical Center	14	16%	0	0%	15	30%
Columbia University Medical Center	15	18%	5	6%	5	10%
**Age (year), median (IQR)**	70.0 (62.5-75.6)		64.8 (55.9-70.9)		65.4 (54.7-72.2)	
**Sex**						
Male	44	51%	44	51%	33	66%
Female	42	49%	42	49%	17	34%
**Race**						
White	81	94%	75	87%	42	84%
Black/African-American	0	0%	4	5%	5	10%
Asian	1	1%	1	1%	0	0%
Other	4	5%	6	7%	3	6%
**Smoking status**						
Current smoker	4	5%	4	5%	11	22%
Past smoker	44	51%	34	39%	17	34%
Never smoker	38	44%	48	56%	22	44%
**Body-mass index (kg/m^2), median (IQR)**	26.6 (24.0-29.7)		27.5 (24.4-31.6)		25.0 (22.8-27.6)	
**Diabetes**						
No	56	65%	80	93%	23	46%
Yes	30	35%	6	7%	27	54%
**Chronic pancreatitis etiology**						
Alcohol	-	-	-	-	16	32%
Autoimmune	-	-	-	-	2	4%
Congenital variant	-	-	-	-	3	6%
Duct stricture or stones	-	-	-	-	7	14%
Idiopathic	-	-	-	-	21	42%
Other	-	-	-	-	1	2%
**AJCC 8th ed pTNM staging**						
Up-front surgical resection						
T0-2N0M0	13	25%	-	-	-	-
T3-4N0M0	2	4%	-	-	-	-
T1-4N1M0	23	44%	-	-	-	-
T1-4N2M0	14	27%	-	-	-	-
Neoadjuvant treatment						
T0-2N0M0	22	65%	-	-	-	-
T3-4N0M0	1	3%	-	-	-	-
T1-4N1M0	7	20%	-	-	-	-
T1-4N2M0	4	12%	-	-	-	-

Abbreviations: AJCC 8th ed pTNM staging, American Joint Committee on Cancer eighth edition tumor-node-metastasis staging; IQR, interquartile range

Ben-Ami R, *et al. Gut* 2024; 73:639–648. doi: 10.1136/gutjnl-2023-331074

Supplemental Table 6. Patient characteristics and circulating marker results for testing set participants

See supplemental excel file

Supplemental Table 7. Discrimination of early-stage pancreatic cancer patients and healthy controls by multi-marker panels in the testing and validation sets

**Biomarkers**	**No. Cases**	**No. Cntrls**	**AUC (95% CI)**
Testing Set			
CA19-9	84	82	0.88 (0.83-0.94)
CA19-9 + TIMP1 + LRG1	84	82	0.94 (0.90-0.97)
CA19-9 + TIMP1 + 9-loci cfDNA methylation panel *	84	82	0.94 (0.90-0.97)
CA19-9 + LRG1 + 9-loci cfDNA methylation panel *	84	82	0.94 (0.90-0.97)
CA19-9 + TIMP1 + LRG1 + 9-loci cfDNA methylation panel *	84	82	0.94 (0.91-0.98)
Validation set			
CA19-9	40	40	0.82 (0.72-0.92)
CA19-9+ TIMP1 + LRG1	40	40	0.82 (0.72-0.92)
CA19-9 + TIMP1 + 9-loci cfDNA methylation panel *	40	40	0.86 (0.77-0.95)
CA19-9 + LRG1 + 9-loci cfDNA methylation panel *	40	40	0.79 (0.68-0.90)
CA19-9 + TIMP1 + LRG1 + 9-loci cfDNA methylation panel *	40	40	0.83 (0.73-0.93)

Abbreviations: AUC, area under the receiver-operator characteristic curve; cfDNA, cell-free DNA; PDAC, pancreatic ductal adenocarcinoma

* 9-loci cfDNA methylation panel that includes nine exocrine pancreas loci encompassing 61 CpG sites

Supplemental Table 8. Clinical characteristics of pancreatic cancer cases and matched controls in the validation set

Characteristics	PDAC cases (N=40)		Healthy Controls (N=40)	
	No.	%	No.	%
**Age (year), median (IQR)**	69.8 (62.6-75.3)		69.8 (61.4-73.2)	
**Sex**				
Male	17	43%	17	43%
Female	23	58%	23	58%
**Race**				
White	34	85%	35	88%
African American	5	13%	4	10%
Asian	1	3%	1	3%
**Ethnicity**				
Hispanic Latino	0	0%	1	3%
Not Hispanic or Latino	40	100%	39	98%
**Tobacco Use**				
Current smoker	1	3%	-	-
Past smoker	19	50%	-	-
Never smoker	20	48%	-	-
**BMI (kg/m^2), median (IQR)**	27.5 (25.4-31.5)			
**Type 2 diabetes**				
No	28	70%	-	-
Yes	12	30%	-	-
**AJCC 8th^{ed} pTNM staging***				
Up-front surgical resection				
T0-2N0M0	7	18%	-	-
T3-4N0M0	2	5%	-	-
T1-4N1M0	9	23%	-	-
T1-4N2M0	9	23%	-	-
TxNxM1	1	3%		
Neoadjuvant treatment				
T0-2N0M0	3	8%	-	-
T3-4N0M0	1	2%	-	-
T1-4N1M0	3	8%	-	-
T1-4N2M0	2	5%	-	-
TxNxM1	3	8%		
**Surgical Resection Status**				
Resected	32	80%	-	-
Aborted for vascular involvement	4	10%	-	-
Aborted for intra-operative metastases	4	10%	-	-
**Neoadjuvant Treatment**				
No	31	78%	-	-
Yes	9	23%	-	-

Abbreviations: IQR, interquartile range; PDAC, pancreatic ductal adenocarcinoma

* American Joint Committee on Cancer 8th edition staging

Supplemental Table 9. Patient characteristics and circulating marker results for validation set participants

See supplemental excel file.

Ben-Ami R, *et al. Gut* 2024; 73:639–648. doi: 10.1136/gutjnl-2023-331074

Supplemental Table 10. Discrimination of pancreatic cancer patients compared with healthy controls for individual protein and cell-free DNA methylation markers in the testing and validation sets

Circulating marker	Testing Set				Validation Set		
	No. Cases	No. Cntrls	AUC (95% CI)		No. Cases	No. Cntrls	AUC (95% CI)
CA19-9	86	86	0.89 (0.84-0.94)		40	40	0.82 (0.72-0.92)
TIMP1	86	86	0.83 (0.77-0.89)		40	40	0.76 (0.65-0.87)
LRG1	86	86	0.79 (0.73-0.86)		40	40	0.56 (0.43-0.69)
9-loci cfDNA methylation panel*	84	82	0.69 (0.61-0.77)		40	40	0.69 (0.58-0.81)

Abbreviations: AUC, area under the receiver-operator characteristic curve; cfDNA, cell-free DNA; PDAC, pancreatic ductal adenocarcinoma

* cfDNA methylation assay that includes nine exocrine pancreas loci encompassing 61 CpG sites

BMJ Publishing Group Limited (BMJ) disclaims all liability and responsibility arising from any reliance placed on this supplemental material which has been supplied by the author(s)

Supplemental Figures

Supplemental Figure 1. Methylation patterns of pancreas-specific cfDNA markers in pancreatic cancer.

Supplemental Figure 2. Heat map of methylation status of pancreas markers (CpGs covered by Illumina Infinium HumanMethylation450 BeadChip Array) in multiple types of primary cancers

Supplemental Figure 3. Circulating cell-free DNA detection for pancreatic cancer cases and controls in training set

Supplemental Figure 1. Methylation patterns of pancreas-specific cfDNA markers in pancreatic cancer. CpGs covered by Illumina Infinium HumanMethylation450 BeadChip Array in acinar, ductal cells and whole pancreas, extracted from results of PCR amplification and sequencing of the markers (that originally are composed of 4< CpG sites), and the average methylation status of the CpGs in 8 whole pancreas normal tissue and 127 primary pancreatic tumors, downloaded from the TCGA portal.

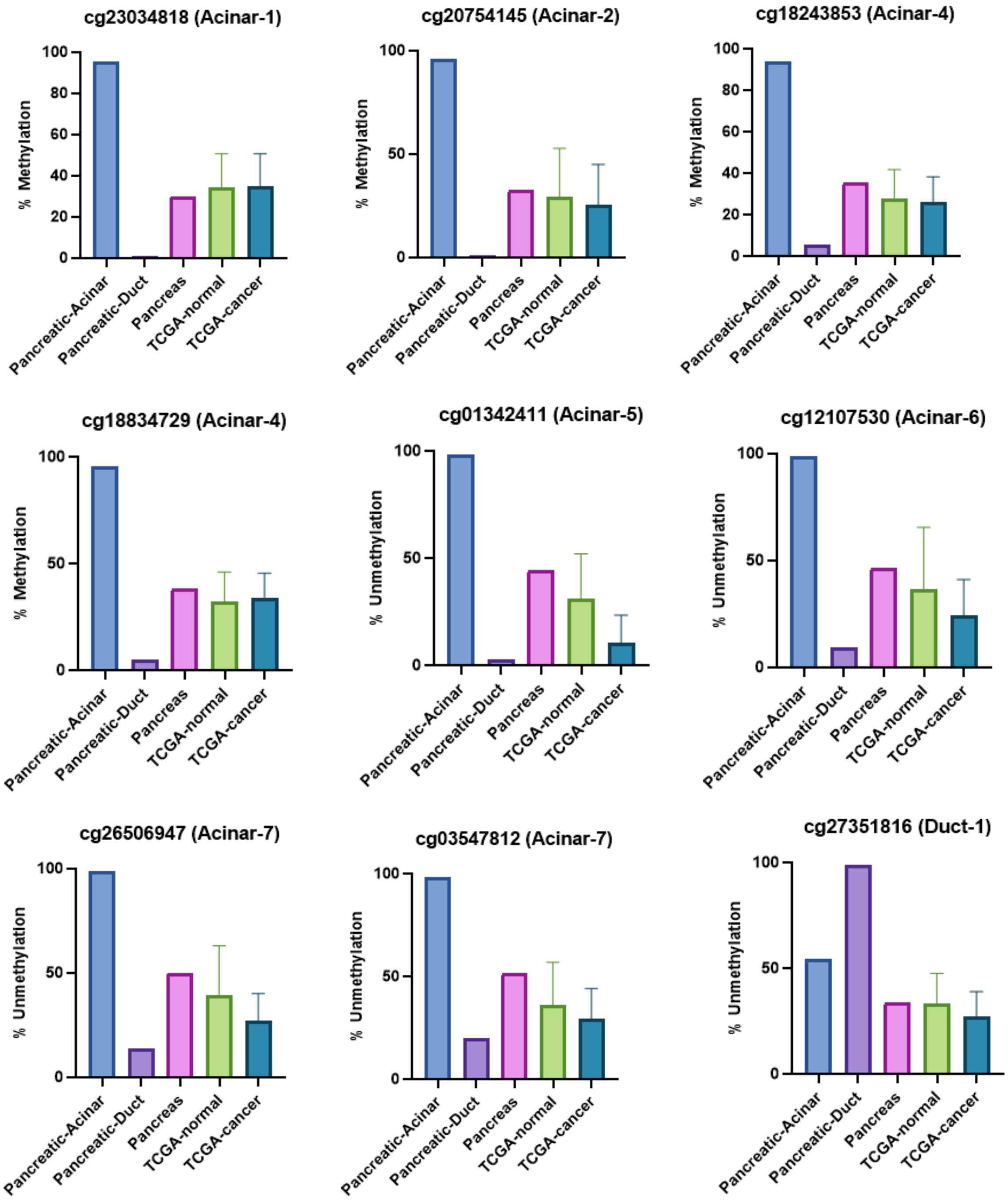

Ben-Ami R, *et al. Gut* 2024; 73:639–648. doi: 10.1136/gutjnl-2023-331074

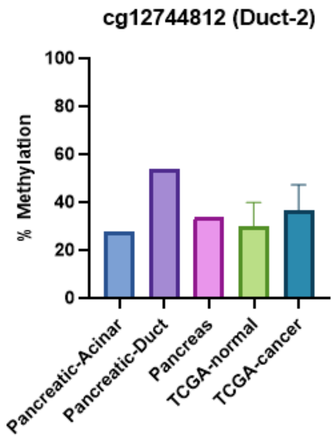
cg12744812 (Duct-2)
% Methylation
100
80
60
40
20
0
Pancreatic-Acinar
Pancreatic-Duct
Pancreas
TCGA-normal
TCGA-cancer

3

Supplemental Figure 2. Heat map of methylation status of pancreas markers (CpGs covered by Illumina Infinium HumanMethylation450 BeadChip Array) in multiple types of primary cancers.

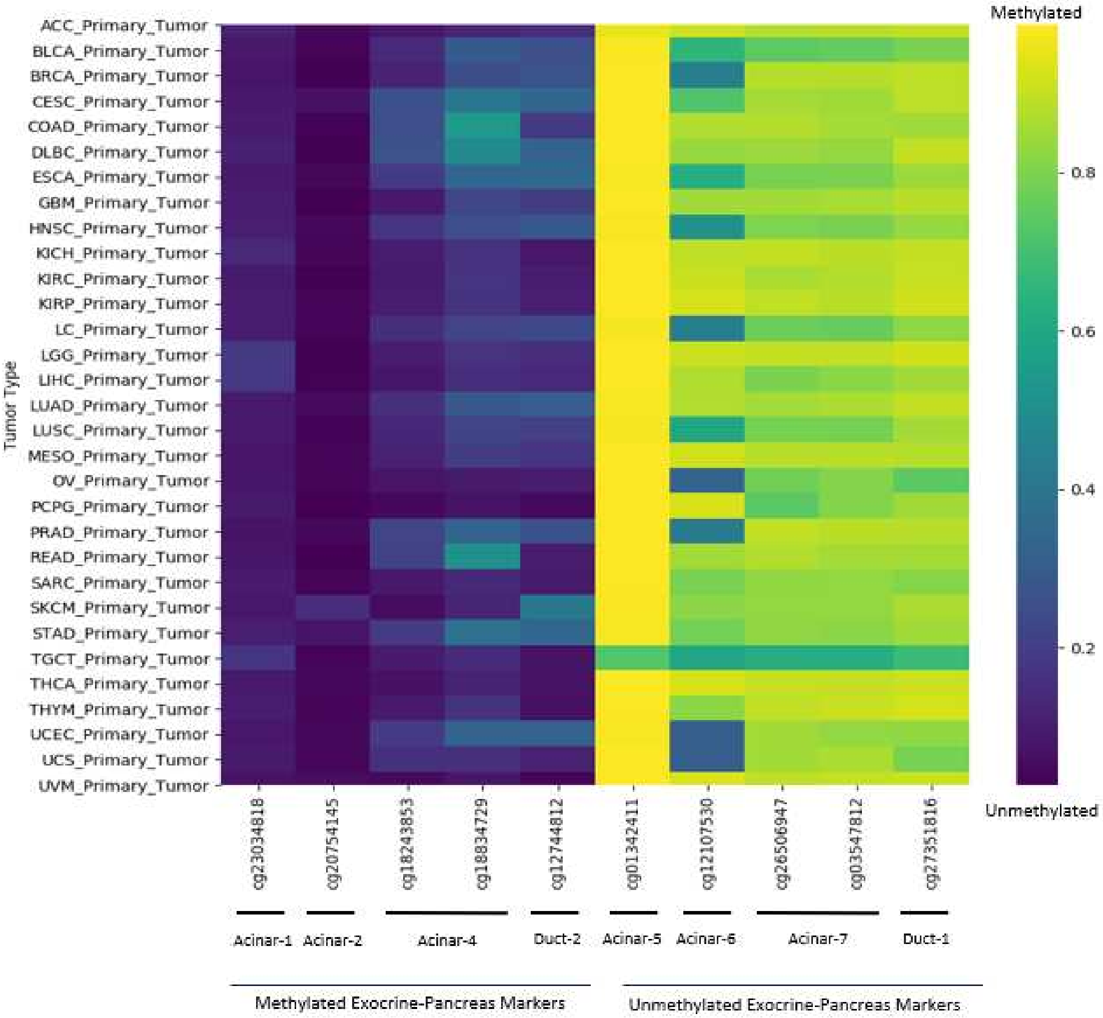

ACC- Adrenocortical Carcinoma, **BLCA** – Bladder Urothelial Carcinoma**,** **BRCA** – Breast invasive Carcinoma, **CESC** – cervical squamous Cell Carcinoma and Endocervical Adenocarcinoma, **COAD** – Colon Adenocarcinoma, **DLBC** – Diffuse Large B-Cell Lymphoma, **ESCA** – Esophageal Carcinoma, **GBM** – Glioblastoma Multiforme, **HNSC** – Head and Neck Squamous Cell Carcinoma, **KICH** – Kidney Chromophobe, **KIRC** – Kidney Renal Clear Cell Carcinoma, **KIRP** – Kidney Renal Papillary Cell Carcinoma, **LC** – Liver Cholangiocarcinoma, **LGG** – Brain Lower-Grade Glioma, **LIHC** – Liver Hepatocellular Carcinoma, **LUAD** – Lung Adenocarcinoma, **LUSC** – Lung Small Cell Carcinoma, **MESO** – Mesothelioma, **OV** – Ovarian Serous Cystadenocarcinoma, **PCPG** – Pheochromocytoma and Paraganglioma, **PRAD** – Prostate Adenocarcinoma, **READ** – Rectum Adenocarcinoma, **SARC** – Sarcoma, **SKCM** – Skin Cutaneous Melanoma, **STAD** – Stomach Adenocarcinoma, **TGCT** – Testicular Germ Cell Tumors, **THCA** – Thyroid Carcinoma, **THYM** – Thymoma, **UCEC** – Uterine Corpus Endometrial Carcinoma, **UCS** – Uterine Carcinosarcoma, **UVM** – Uveal Melanoma.

Data downloaded from TCGA portal.

Ben-Ami R, *et al. Gut* 2024; 73:639–648. doi: 10.1136/gutjnl-2023-331074

Supplemental Figure 3. Circulating cell-free DNA detection for pancreatic cancer cases and controls in training set

(A) Circulating cfDNA *KRAS* mutation detection in training set

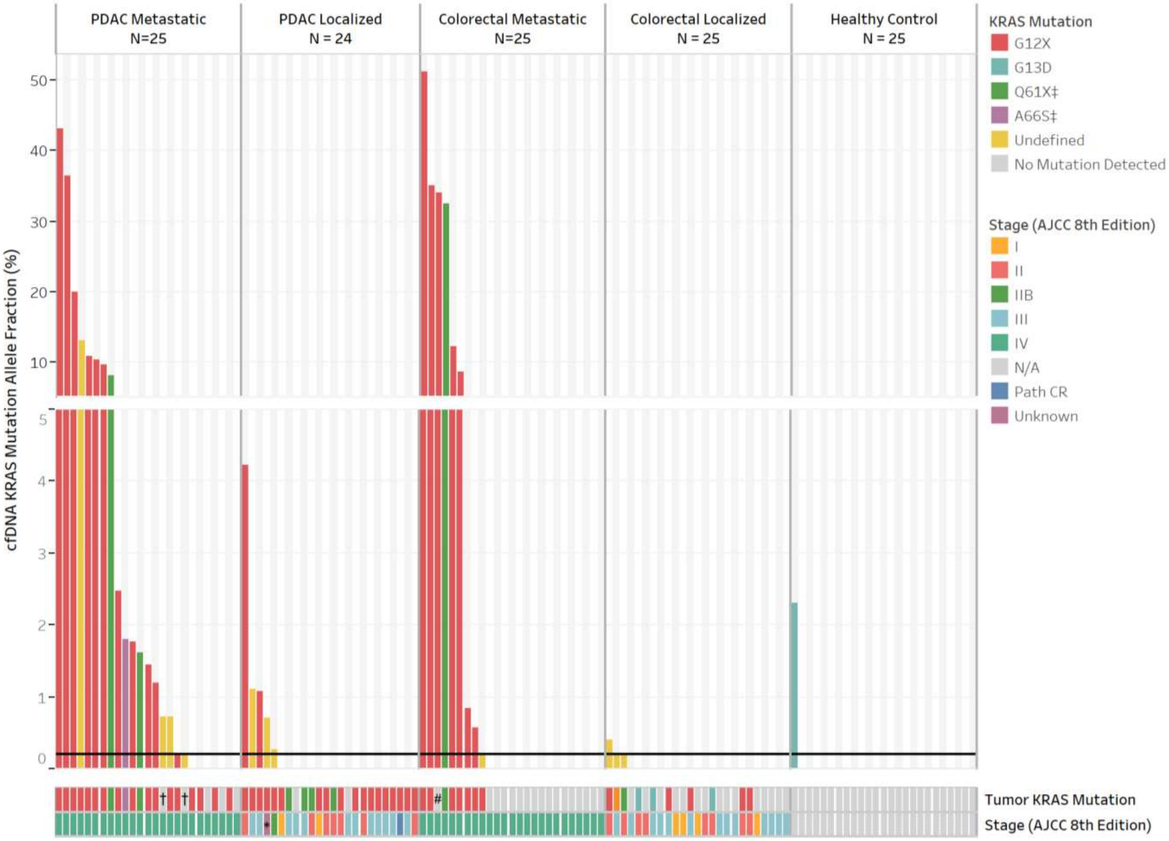

Abbreviations: AJCC, American Joint Committee on Cancer; cfDNA, cell-free DNA; Path CR, pathologic complete response; PDAC, pancreatic ductal adenocarcinoma

Vertical bars represent *KRAS* mutation allele fraction in plasma cfDNA. Horizontal black line indicates cutoff for positive assay (≥0.2% allele fraction). Corresponding tumor characteristics in tracks below.

† *KRAS* mutation detected in cfDNA but not in CLIA-certified NGS of tumor tissue. Manual review of tumor tissue indicated low tumor cellularity and *KRAS* mutation present at low allele fraction in tumor.

# Tumor cellularity was inadequate for somatic DNA extraction and sequencing.

* Patient had a localized tumor that was surgically resectable. Due to comorbid conditions, did not undergo surgery and thus pathologic staging was not available.

‡ Determined via NGS. KRAS p.G12X and p.G13D determined via multiplexed droplet digital PCR.

Ben-Ami R, *et al. Gut* 2024; 73:639–648. doi: 10.1136/gutjnl-2023-331074

(B) Exocrine pancreas-specific methylation marker detection in training set

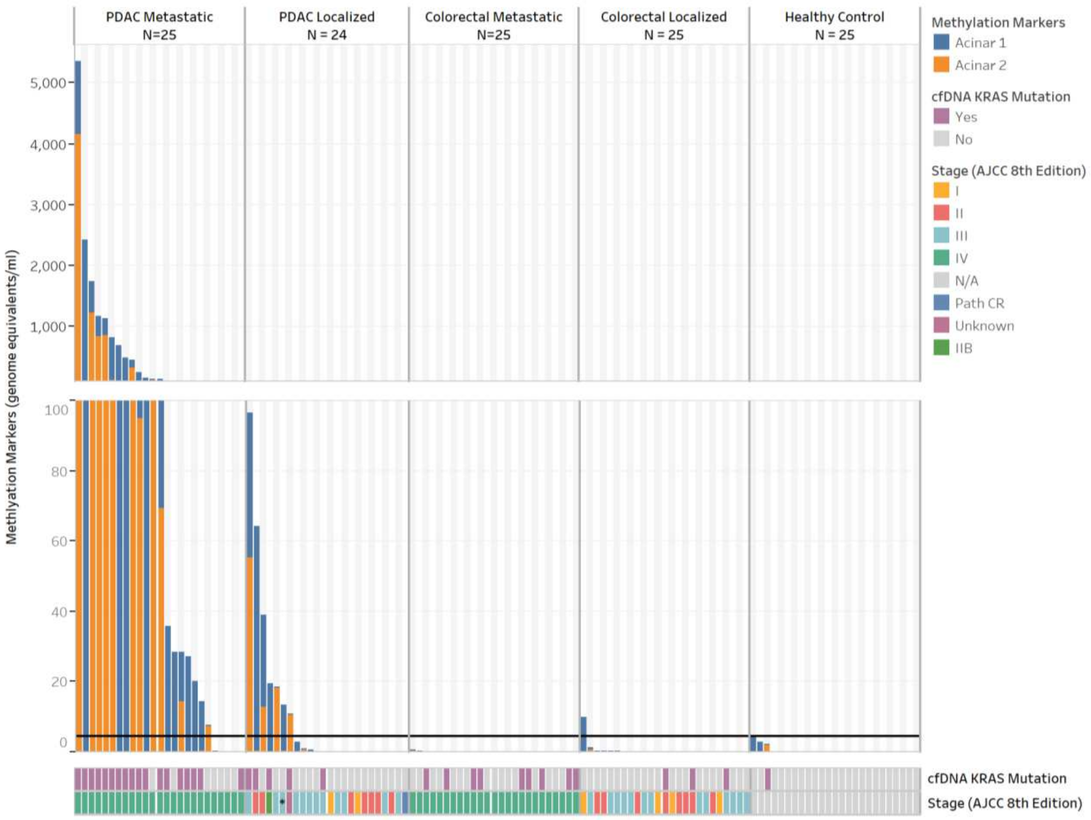

Abbreviations: AJCC, American Joint Committee on Cancer; cfDNA, cell-free DNA; Path CR, pathologic complete response; PDAC, pancreatic ductal adenocarcinoma

Vertical bars represent genome equivalents per mL of methylated cfDNA fragments in plasma. Horizontal black line indicates cutoff for positive assay, as determined to provide >98% specificity (≥4.5 genome equivalents/ml). Corresponding patient characteristics in tracks below.

* Patient had a localized tumor that was surgically resectable. Due to comorbid conditions, did not undergo surgery and thus pathologic staging was not available.

Ben-Ami R, *et al. Gut* 2024; 73:639–648. doi: 10.1136/gutjnl-2023-331074

REFERENCES

1 Oxnard GR, Paweletz CP, Kuang Y, *et al.* Noninvasive detection of response and resistance in egfrmutant lung cancer using quantitative next-generation genotyping of cell-free plasma DNA. *Clin Cancer Res* 2014;**20**:1698–705. doi:10.1158/1078-0432.CCR-13-2482

2 Moss J, Magenheim J, Neiman D, *et al.* Comprehensive human cell-type methylation atlas reveals origins of circulating cell-free DNA in health and disease. *Nat Commun* 2018;**9**. doi:10.1038/s41467-018-07466-6

3 Lehmann-Werman R, Neiman D, Zemmour H, *et al.* Identification of tissue-specific cell death using methylation patterns of circulating DNA. *Proc Natl Acad Sci U S A* 2016;**113**:E1826–34. doi:10.1073/pnas.1519286113

4 Zemmour H, Planer D, Magenheim J, *et al.* Non-invasive detection of human cardiomyocyte death using methylation patterns of circulating DNA. *Nat Commun* 2018;**9**. doi:10.1038/s41467-018-03961-y

5 Neiman D, Gillis D, Piyanzin S, *et al.* Multiplexing DNA methylation markers to detect circulating cell-free DNA derived from human pancreatic β cells. *JCI Insight* 2020;**5**. doi:10.1172/JCI.INSIGHT.136579

6 Garcia EP, Minkovsky A, Jia Y, *et al.* Validation of oncopanel a targeted next-generation sequencing assay for the detection of somatic variants in cancer. *Arch Pathol Lab Med* 2017;**141**:751–8. doi:10.5858/arpa.2016-0527-OA

7 MacConaill LE, Campbell CD, Kehoe SM, *et al.* Profiling critical cancer gene mutations in clinical tumor samples. *PLoS One* 2009;**4**. doi:10.1371/journal.pone.0007887
